# Genetic diversity analysis and variety identification using SSR and SNP markers in melon

**DOI:** 10.1186/s12870-023-04056-7

**Published:** 2023-01-18

**Authors:** Jian Zhang, Jingjing Yang, Yanling Lv, Xiaofei Zhang, Changxuan Xia, Hong Zhao, Changlong Wen

**Affiliations:** 1 Beijing Vegetable Research Center (BVRC), Beijing Academy of Agricultural and Forestry Sciences, National Engineering Research Center for Vegetables, Beijing, 100097 China; 2 Beijing Key Laboratory of Vegetable Germplasms Improvement, Beijing, 100097 China; 3 Institute of Vegetable, Liaoning Academy of Agricultural Sciences, Shenyang, 110161 China

**Keywords:** Perfect SSR, Perfect SNP, Genetic diversity, Variety identification, Melon

## Abstract

Melon is an important horticultural crop with a pleasant aromatic flavor and abundance of health-promoting substances. Numerous melon varieties have been cultivated worldwide in recent years, but the high number of varieties and the high similarity between them poses a major challenge for variety evaluation, discrimination, as well as innovation in breeding. Recently, simple sequence repeats (SSRs) and single nucleotide polymorphisms (SNPs), two robust molecular markers, have been utilized as a rapid and reliable method for variety identification. To elucidate the genetic structure and diversity of melon varieties, we screened out 136 perfect SSRs and 164 perfect SNPs from the resequencing data of 149 accessions, including the most representative lines worldwide. This study established the DNA fingerprint of 259 widely-cultivated melon varieties in China using Target-seq technology. All melon varieties were classified into five subgruops, including ssp. *agrestis*, ssp. *melo*, muskmelon and two subgroups of foreign individuals. Compared with ssp. *melo*, the ssp. *agrestis* varieties might be exposed to a high risk of genetic erosion due to their extremely narrow genetic background. Increasing the gene exchange between ssp. *melo* and ssp. *agrestis* is therefore necessary in the breeding procedure. In addition, analysis of the DNA fingerprints of the 259 melon varieties showed a good linear correlation (R^2^ = 0.9722) between the SSR genotyping and SNP genotyping methods in variety identification. The pedigree analysis based on the DNA fingerprint of ‘Jingyu’ and ‘Jingmi’ series melon varieties was consistent with their breeding history. Based on the SNP index analysis, ssp. *agrestis* had low gene exchange with ssp. *melo* in chromosome 4, 7, 10, 11and 12, two specific SNP loci were verified to distinguish ssp. *agrestis* and ssp. *melon* varieties. Finally, 23 SSRs and 40 SNPs were selected as the core sets of markers for application in variety identification, which could be efficiently applied to variety authentication, variety monitoring, as well as the protection of intellectual property rights in melon.

**Supplementary Information:**

The online version contains supplementary material available at 10.1186/s12870-023-04056-7.

## Introduction

Melon (*Cucumis melo* L.), an important horticultural crop within the Cucurbitaceae family, has a pleasant aromatic flavor and is also a rich source of soluble sugars, minerals, organic acids, vitamins, and other health-promoting substances [1]. It is thought to have originated in Africa and Asia and exhibits major natural variation around the world [2]. Currently, China ranks first globally for melon cultivation, the yield of which in China was 16 million tons and accounted for more than half of the global output (31.2 million, 51.3%) in 2019 (FAO http://faostat.fao.org/). As a result of the continuous improvement of melon varieties, increasing numbers of melon varieties have been created and sold in China, including melon cultivars introduced from foreign countries [3]. China has also become the largest market for melon seeds globally, and numerous melon varieties have been submitted for registration to date. This poses a major challenge for variety evaluation, discrimination, and innovation in breeding. The current standard of melon variety identification is based on field inspection, which is laborious and easily affected by the environment. Compared with phenotypic identification method in the field, DNA fingerprinting using molecular markers has proved to be fast, accurate, and free from the influence of the environment throughout the growth period in varieties identification [4]. In past years, the genetic diversity of melon varieties was little revealed whereas several reports focused on the melon germplasm [3–6].

Over the past decades, several studies have assessed the classification of melon subspecies as well as the genetic diversity of melon using simple sequence repeat (SSR) and single nucleotide polymorphism (SNP) markers [3–6]. However, primary focus has been the phylogenetic relationships among germplasms or breeding lines rather than commercial varieties. Moreover, researchers have often encountered low polymorphism and a low success rate in SSR and SNP genotyping. It has been reported that just 52 polymorphic SSRs were selected from a total set of 1219 SSRs in melon and only 23 SSRs were selected as highly polymorphic SSRs in the 995 SSR markers in cucumber [7]. One study detected a total of 52 SNP loci for melon variety discrimination, while only two SNPs were converted into CAPS markers for practical use [8]. The reason for this is probably the lack of use of perfect SSRs/SNPs, for which motifs and variations are clear and steady and for which there are flanking sequences [9, 10]. With the development of high-throughput sequencing, the genome of melon and numerous melon accessions have been sequenced and characterized using the next-generation sequence platform providing a foundation for the selection of perfect SSRs/SNPs in melon [5, 11–13]. In earlier studies, genome-wide perfect SSRs and SNPs were efficiently established and evaluated for variety identification in cucumber [10, 14], eggplant [15], pepper [16], and watermelon [17]. Therefore, the application of DNA fingerprinting in melon varieties using genome-wide perfect SSRs and SNPs is reasonable.

Previous studies have revealed that melon mainly consists of two populations, ssp. *melo* (thick-skinned melon) and ssp. *agrestis* (thin-skinned melon) [13]. These two populations can be easily distinguished by fruit morphology, but identifying them at the seedling stage is highly challenging, especially for hybrid varieties of these two populations. To study the genetic diversity and pedigree of the dominant melon varieties in China, we screened out genome-wide perfect SSRs and perfect SNPs from 149 re-sequenced accessions and established the DNA fingerprint of 259 melon varieties with 136 perfect SSRs and 164 perfect SNPs using Target-seq technology. Five subpopulations were observed, including ssp. *agrestis*, ssp. *melo*, muskmelon and two subgroups of foreign individuals. The DNA fingerprinting results of the 259 melon varieties identified by SSR markers were linearly correlated to that of the SNP markers between pairs of varieties. We obtained two specific SNP loci used to identify the ssp. *agrestis* varieties. Finally, a core set of 23 SSRs and 40 SNPs was selected for use in variety supervision, identification, and the protection of intellectual property rights in melon varieties.

## Materials and methods

### Plant material and DNA isolation

A total of 259 melon varieties with diverse morphological characteristics were used in this study. This panel consisted of all ecological types and primary melon varieties cultivated in China, including 125 commercial varieties from Chinese seed market, 28 foreign hybrid varieties from foreign countries, 84 landraces, and 22 breeding lines from the Beijing Vegetable Research Center (BVRC) germplasm bank (Additional file 1: Table S1). For the Target-seq of this melon collection, genomic DNA was isolated using young leaf tissues from 30 seeds according to a modified sodium dodecyl sulfate (SDS) method [18]. The quantity and quality of the DNA in each sample were measured by a NanoDrop 2000 spectrophotometer (ThermoFisher Scientific, USA). The final concentration of the DNA was adjusted to 100 ng/μL for Target-seq technology.

### Discovery of genome-wide perfect SSRs and SNPs based on the melon variome

The resequencing data of 149 melon accessions downloaded from the NCBI SRA (PRJNA565104) were used for the discovery of genome-wide perfect SSRs and SNPs. These 149 accessions were selected from 1175 accessions based on the phylogenetic tree and represent the global diversity of the species (Additional file 1: Table S2), of which 1175 re-sequenced accessions were reported in a previous study [12]. First, the melon reference genome (DHL92 v.3.5.1) was analyzed to identify genome-wide SSRs by GMATA with previously described parameters [19]. The SSR loci with unique 2–6-bp motifs and a motif length less than 50 bp were screened out. Secondly, the resequencing data of 149 melon lines were aligned to the observed SSRs, and strict filtering was applied as follows: no SNP, Indel, or other SSR in the SSR motif and the associated 50-bp flanking sequence, and a reads frequency of the major SSR allele in each accession of more than 0.7. The perfect SNPs had to meet the following criteria: (i) missing rate < 0.1; (ii) heterozygosity < 0.1; (iii) minor allele frequency (MAF) > 0.05; (iv) the 50-bp flanking sequence of SNP was unique to the melon genome; (v) no other variation in the flanking sequence of 50 bp. Finally, evenly-distributed perfect SSRs and SNPs were sent for multiplex PCR panel design at Molbreeding Biotechnology Company (Shijiazhuang, China), and the DNA of 259 melon varieties was genotyped by Target-seq technology as previously reported [10, 14].

### Genetic diversity analysis in melon varieties

The genetic information such as the polymorphic information content (PIC), heterozygosity of loci and samples (He), and the missing rate were calculated in Excel 2016 (Microsoft Corp., Redmond, USA). The population structure of the 259 melon individuals was analyzed by all SSR and SNP genotypes in STRUCTURE v2.3 for K values ranging from 1 to 10. Each running was repeated 5 times with 50,000 burn-in length and 10,000 MCMC iterations. The most likely population ancestor was depended on △K [20, 21]. Varieties with less than 70% membership in a certain population are considered admixed population. The neighbor-joining tree among the 259 varieties was established using the poppr R package with Bruvo’s genetic distance [22] and was visualized by MEGA7 [23]. To investigate population differentiation, an analysis of molecular variance (AMOVA) among groups was calculated in R [24].

### Selection of population-specific SNP markers in melon

In China, melon varieties are generally divided into two major groups, namely ssp. *agrestis* and ssp. *melo*, for which there are major differences in cultivation and commercial use [25]. To implement population classification at the DNA level, we attempted to select population-specific SNP markers that differentiated the ssp. *agrestis* and ssp. *melo*. First, we calculated the SNP frequency of 86 melon ssp. *agrestis* and 58 ssp. *melo* in all perfect SNP loci. Then the SNP-index across 12 chromosome was calculated using sliding widow analysis method (window size 100 kb and step size 10 kb). Finally, we obtained the specific SNP loci used for population discrimination and verified in KASP platform.

### Core set of SSR and SNP markers for melon variety determination

According to variety identification using UPOV [26], two varieties are believed to be identical when they have the same genotype in a certain set of molecular markers. The core set of SSRs or SNPs aims to select the minimum number of markers that can efficiently discriminate the maximal number of varieties. An in-house Perl program was used to calculate the core set of SSRs and SNPs for differentiation of the melon varieties (Additional file 2), which had successfully used in cucumber, pepper and watermelon varieties identification [10, 14, 16]. In addition, a diagonal matrix was calculated by counting the numbers of different markers for each pair of DNA samples among the 259 melon varieties. The variety identification results using SSRs were compared with the results of the SNP-based method.

## Results

### Discovery of genome-wide perfect SSRs/SNPs in melon

**Fig. 1:**
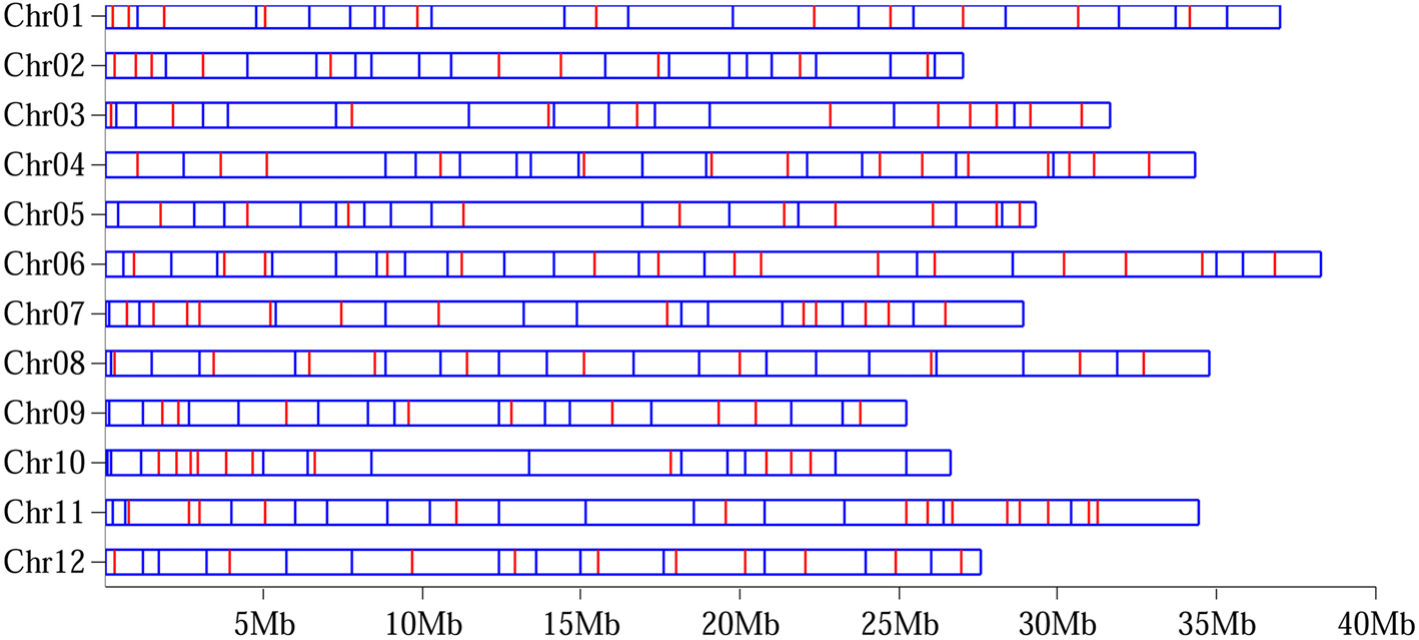
Distribution of 136 perfect SSRs and 164 perfect SNPs on the 12 chromosomes in the melon genome. The red lines indicate the SSR loci and the blue lines indicate the SNPs

To discover the genome-wide perfect SSRs and SNPs, the sequence reads of the 149 melon accessions were aligned to the reference genome DHL92 v.3.5.1. We identified a total of 67,782 SSRs and 5,520,726 SNPs, with an average of 167 SSRs per Mb and 13.6 SNPs per kb on melon genome [27]. According to the screening criteria of perfect SSRs and SNPs, we obtained 17,190 (25.4%) perfect SSRs and 293,320 (5.3%) perfect SNPs (Additional file 1: Table S3 and Table S4). Finally, we selected 136 perfect SSRs and 164 perfect SNPs to establish the DNA fingerprints of the 259 melon varieties by Target-seq technology. These perfect SSRs/SNPs had a high correlation (0.98) with the genome-wide perfect SSRs/SNPs based on the genetic distance matrix of 149 melon accessions (Additional file 1: Table S5). The number of SSRs ranged from 9–15 per chromosome, while the value of the SNPs was 11 to 16 per chromosome. The average physical distance between two adjacent SSR and SNP markers was 2.57 Mb and 2.23 Mb, respectively (Fig. 1).

### Genetic diversity of the 259 melon varieties

Based on the Target-seq results, we obtained the fingerprints of 259 melon varieties in 136 perfect SSR and 164 perfect SNP loci (Additional file 3). A total of 106.3 million short sequence reads were generated on 78,218 data points, with an average depth of 1358.8-fold for genotype. Furthermore, 95% amplicons were centralized in an ideal sequence depth (500–5000 ×), which ensured that most of the genotype data had sufficient coverage and avoided polarized sequencing in other amplicons [28]. In terms of the ratio of major and minor allelic reads, 93.2% of homozygous genotypes were above 0.9 or below 0.1, while 91.6% of heterozygous genotypes were between 0.3 and 0.7.

The number of alleles per SSR locus ranged from 2–15, with an average of 5.3. The PIC value of the SSR loci ranged from 0.11–0.81, while this value in the SNPs ranged from 0.14–0.50 (Additional file 1: Table S6, Additional file 4: Fig. S1a). The missing rate of the genotyped SSRs and SNPs ranged from 0–0.29 and 0–0.06, respectively (Additional file 1: Table S6, Additional file 4: Fig. S1b). The average heterozygosity was 0.20 for the SSR loci and 0.24 for the SNP loci (Additional file 1: Table S6, Additional file 4: Fig. S1c). Interestingly, the heterozygosity of 140 (54.0%) varieties was below 0.1 (Additional file 4: Fig. S1d), indicating that the 259 melon varieties had high similarity and low diversity. In addition, there were nine SSR and six SNP loci that were removed due to their low PIC value and high missing rate.

### Population structure of the melon varieties

 Population structure of the 259 melon varieties was inferred using a subset of 129 SSRs and 159 SNPs performed in the software STRUCTURE v2.3. Phylogenetic tree based on sequence variations also exhibited distinctive separation. The 259 melon varieties were divided into two strong classifications according to the △K value from the structure output (Additional file 4: Fig. S2).

**Fig. 2:**
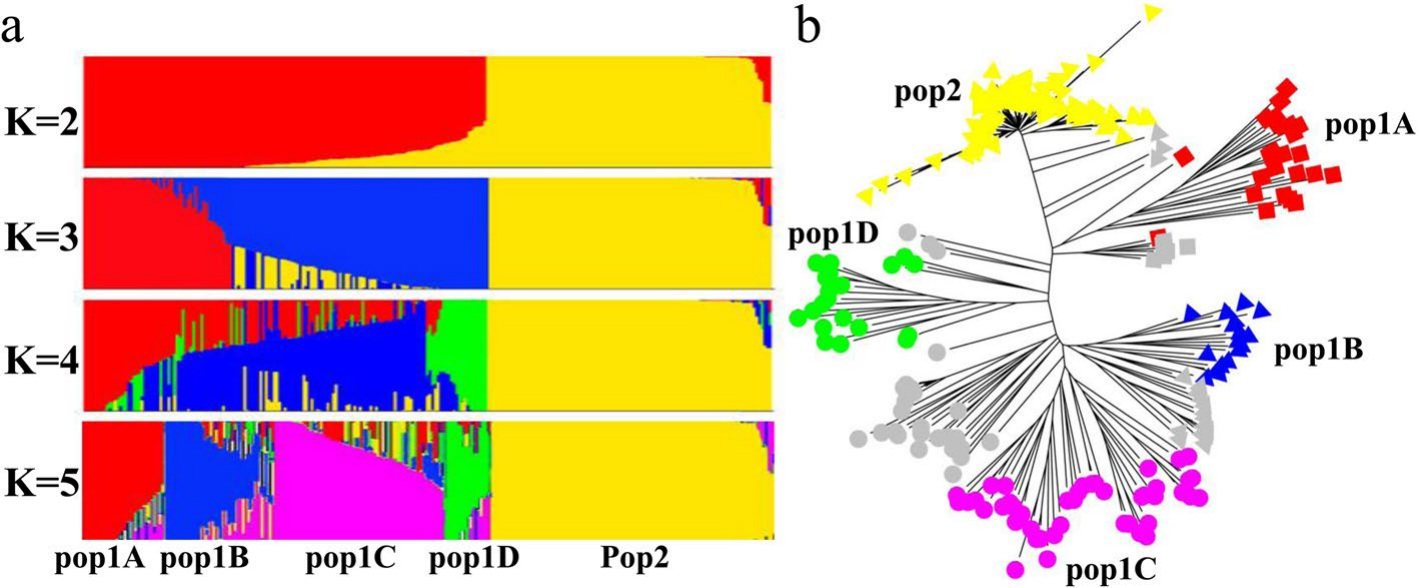
Population structure of the 259 melon varieties. **a** Population structure analysis when K equals 2, 3, 4, and 5. (**b**) Neighbor-joining tree in the five subgroups; pop1A is colored in red, pop1B is colored in blue, pop1C is colored in purple, pop1D is colored in green, pop2 is colored in yellow, and the admixed population is colored in grey

The structure of 259 melon varieties were calculated with K values varying from 2 to 4, as indicated in Fig. 2a. When K = 2, 259 melon varieties are divided into two groups: pop1 with 153 varieties and pop2 with 106 varieties. More than 94.8% melons in pop1 are belong to C. melon ssp. melon, and 97.2% in pop2 are belong to C. melon ssp. *agrestis*. When K = 3, pop 1 was divided into two clusters, one of which was the landraces from foreign countries (pop1A), while the other was the hybrid varieties. When K = 4, the hybrid varieties was separated into two subgroups containing pop1B and pop1C. Pop1B was mainly consisted of muskmelon varieties and the representative varieties in pop1B were ‘Xizhoumi25’ and ‘Jingyumi28’. Compared with pop1B, the genetic relationship of pop1C was close to that of ssp. *agrestis*, which maybe the hybrid between ssp. *agrestis* and ssp. melon., and the representative varieties in pop1C were ‘Yilishabai’, ‘Yinlu1’, and ‘Xiboluotuo’. When K = 5, pop1C generated a new subgroup pop1D which mainly contain foreign hybrid varieties. However, the 103 varieties (97.2%) in pop2 at K = 2 was still in same subgroup when K = 5 (Additional file 1: Table S7). The narrow genetic diversity and close similarity of the ssp. *agrestis* varieties indicate a potential risk of genetic erosion in the breeding process [5].

The pairwise fixation index (*Fst*) among the five melon subgroups ranged from 0.15–0.63 (Additional file 1: Table S8), pop1A, pop1B, pop1C, and pop1D were strongly differentiated from pop2 and the average distance value was 0.58. 206 varieties with 70% membership of each five populations (Pop1A, Pop1B, Pop1C, Pop1D and Pop2) were used for AMOVA analysis. 51.1% of the variation was due to differences among populations, 28.9% was due to differences within populations and 20.1% was due to differences within samples (Table 1). This result indicated a narrow genetic background in the same populations of the 259 melon varieties, but significant differences in different populations. These five subgroups were also in accordance with the neighbor-joining tree performed by MEGA7 (Fig. 2b).

**Table 1:** Analysis of molecular variance (AMOVA) among populations and within populations

Source of variation	Df	Sum of square	Mean square	Variance component	Percentage of variation
Between population	4	26,226.4	6556.6	54.5	51.1
Between samples within population	201	9139.8	45.5	30.8	28.9
Within samples	206	4417.0	21.4	21.4	20.1
Total		39,783.2	96.8	106.8	100

### Comparison of DNA fingerprints between SSR and SNP genotyping methods

**Fig. 3:**
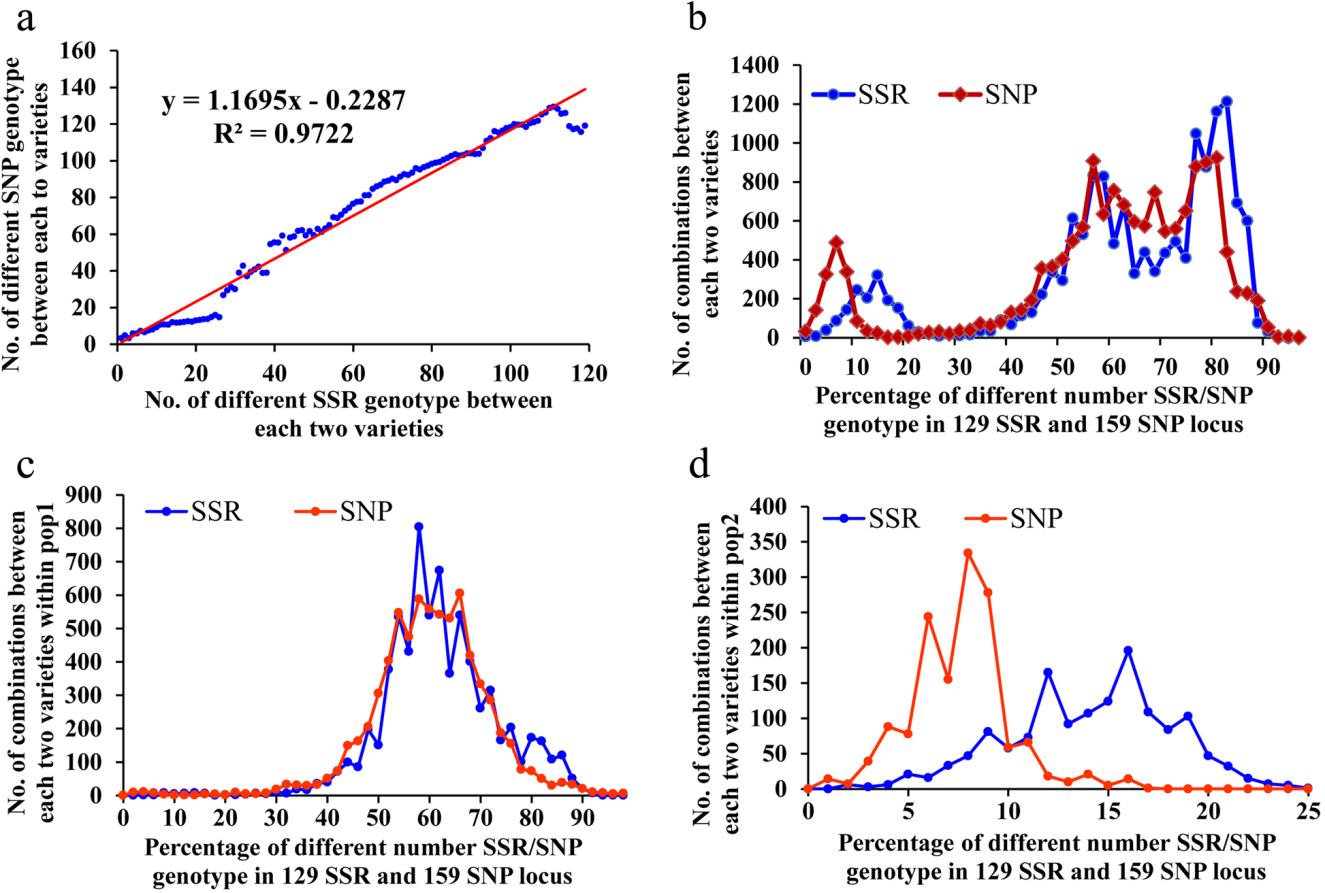
Comparison of variety identification using the SSR and SNP methods. The linear correlation of SSR genotype and SNP genotype in variety identification (**a**). The variation curve of different numbers of SSR genotypes and SNP genotypes between each pair of samples in all (b), pop1(c), and pop2 (d) varieties

Variety identification based on molecular markers was used to compare the genotype between each pair of varieties. The more different the observed SSR/SNP markers were between two varieties, the further the distance of their genetic relationship. To select a core set of SSR and SNP markers for melon variety identification, we counted the number of different genotypes among the 259 melon varieties in 129 perfect SSRs and 159 perfect SNPs. The average of different SSR genotypes between each pair of melon varieties was 82.5 and ranged from 2–121, while the average of different SNP genotypes was 96.4 and ranged from 1–154. The number of different markers in the SSRs was linearly related to the SNPs (R^2^ = 0.9722) in the 259 melon varieties (Fig. 3a).

Because the number of total SSR and SNP markers was not same, we take the percentage of different markers against the total markers as the X-axis, the number of combinations between each two varieties as Y-axis. Interestingly, two peaks were found both in the percentage of different SSR and SNP genotypes with each pair of varieties (Fig. 3b). The number of different markers among the 259 varieties calculated using SSRs was mainly concentrated in less than 20% (25.8 SSR loci) and more than 50% (64.5 SSR loci), while the variation curve of the SNPs was below 10% (12.9 SNP loci) and above 50% (79.5 SNP loci). Different numbers of markers between each pair of varieties exhibited significant differences in pop1 and pop2. For pop1, most varieties had 50% to 80% genotype differences with other varieties in 129 SSR or 159 SNP loci (Fig. 3c). For pop2, the different markers within each pair of varieties respectively had 8 to 16 SNPs and 13 to 26 SSRs, the value of which was much lower than in pop1 (Fig. 3d). The ssp. *agrestis* varieties had a very narrow genetic background compared to the ssp. *melo* varieties. Moreover, the SSR markers were more appropriate for identifying ssp. *agrestis* varieties due to the higher value in the percentage of a different number of genotypes (Fig. 3d). A peak at 80%–90% marker difference for 259 varieties was found, while no peak was observed at this position for pop1 and pop2, indicating few gene exchanges between these two populations.

### Core set of SSR and SNP markers in variety identification

**Fig. 4:**
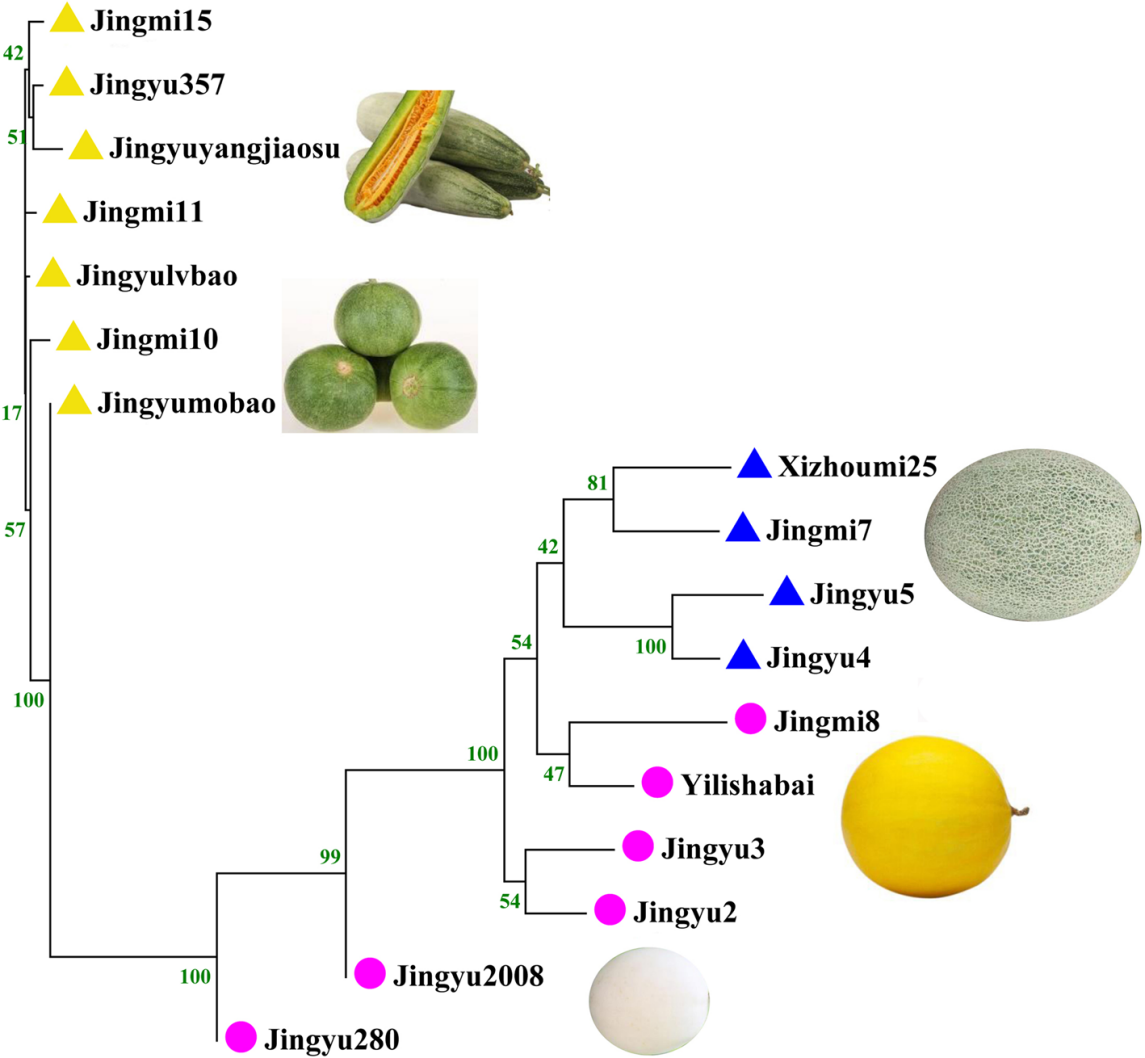
Phylogenetic tree of ‘Jingyu’ and ‘Jingmi’ series varieties. Pop1A is colored in red, pop1B is colored in blue, pop1C is colored in purple, and pop2 is colored in yellow. The symbols for population classification are the same as in Fig. 2

Based on the DNA fingerprinting, we constructed the pedigree of ‘Jingyu’ and ‘Jingmi’ series varieties, which was consistent with the breeding history and population classification (Fig. 4). A Perl script developed in our previous research was used to select the core set of SSRs and SNPs, which distinguished the maximum number of varieties with the minimum number of markers. Finally, we respectively obtained 23 core SSRs and 40 core SNPs that could efficiently differentiate 99% of the 259 melon commercial varieties with at least one different SSR/SNP genotype (Additional file 4: Fig. S3). Each core SSR and SNP loci was also respectively verified by capillary electrophoresis (Additional files 1: Table S9) and kompetitive allele specific (KASP) technology (Additional files 1: Table S10), which are widely used for melon variety identification, and it was proved that the identification time was highly reduced compared with phenotypic identification in the field (24 h vs. several months).

### Population-specific markers of ssp. agrestis and ssp. melo

**Fig. 5:**
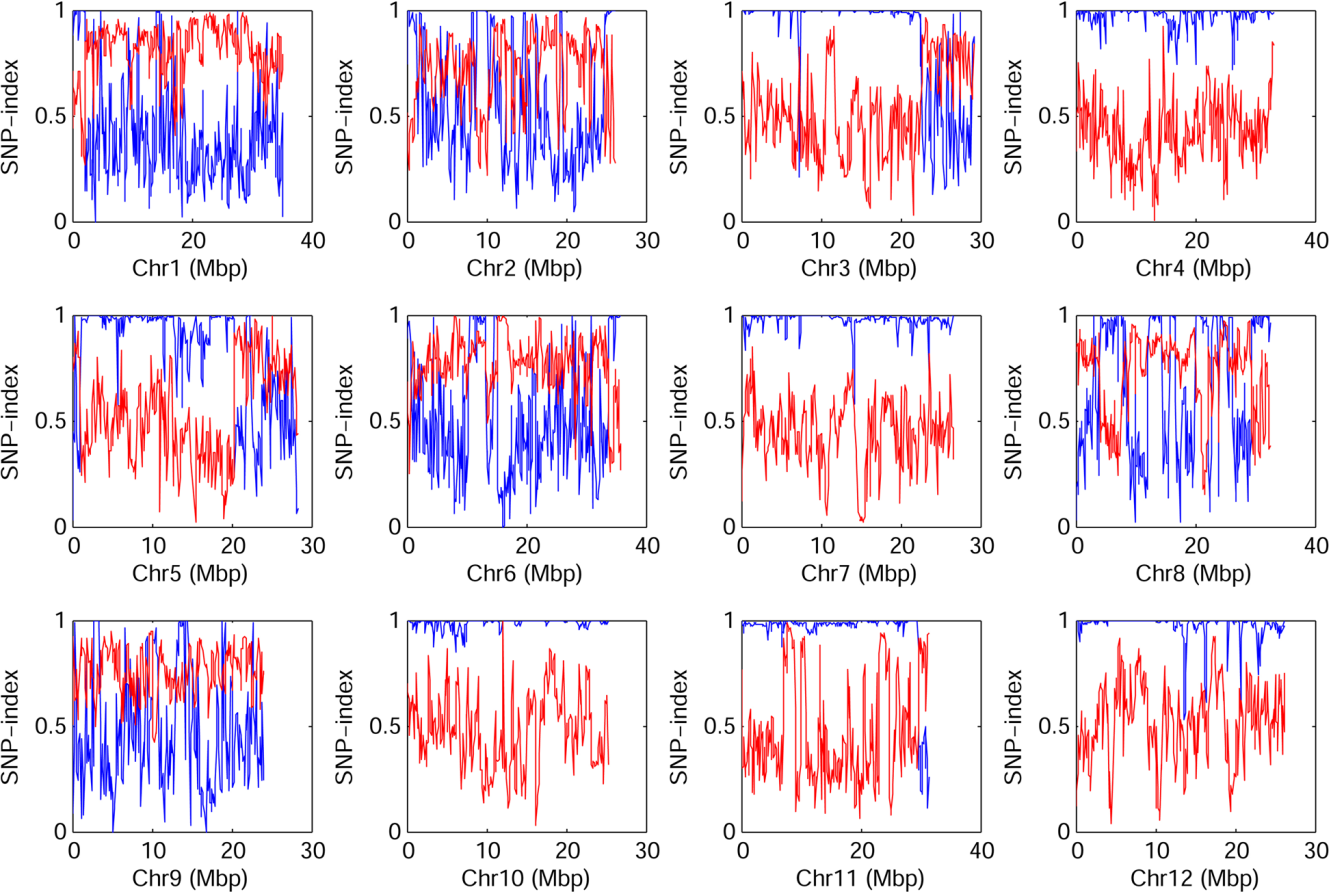
SNP index variation of ssp. *agrestis* and ssp. *melo* across 12 chromosomes. SNP index of ssp. *agrestis* was colored in blue and SNP index of ssp. *melo* was colored in red

The genetic distance of ssp. *melo* and ssp. *agrestis* was far in both germplasm and varieties. Compared with the ssp. *melo* varieties, the ssp. *agrestis* varieties had a very narrow genetic background. Therefore, some conserved SNP alleles may only exist in ssp. *agrestis* varieties, which can be used for population  identification in melon. SNP index of 58 ssp. *melo* and 86 ssp. *agrestis* accessions in 293,320 perfect SNP were calculated (Fig. 5). For ssp. *melo* accessions, the average ratio of reference SNP alleles was 0.598, ranged from 0.418 in chromosome 4 to 0.810 in chromosome 1. For the ssp. *agrestis* accessions, the average SNP-index of reference genome across the 12 chromosome was 0.76, ranged from 0.420 in chromosome 1 to 0.987 in chromosome 10. Moreover, SNP-index of ssp. *agrestis* in chromosome 4, 7, 10, 11and 12 was above 0.95, indicating low gene exchange in these chromosomes. △SNP-index—the ratio of SNP-index between ssp. *melo* and ssp. *agrestis* accessions was calculated, a total of 718 SNP loci obtaining values equal to 1 (Additional file 1: Table. S11), including two loci (MeSNP011and MeSNP047) in Target seq primer panel. For MeSNP011, a total of 104 varieties had the AA allele, and 101 varieties belonged to Pop2 (Additional file 4: Fig. S4a). For MeSNP047, a total of 111 varieties had the TT allele, and 100 varieties belonged to Pop2 (Additional file 4: Fig. S4b). When MeSNP011 was combined with MeSNP047, a total of 101 varieties had the AATT allele, and only one did not belong to Pop2 (Additional file 4: Fig. S4c). Overall, MeSNP011 could identify 98.4% of ssp. *agrestis* varieties and MeSNP047 could distinguish 92.0% of ssp. *agrestis*. These two SNP loci together could identify 99% of ssp. *agrestis* varieties, indicating that these two markers could distinguish ssp. *agrestis* melons.

## Discussion

### Efficient genotyping tools of perfect SSR/SNPs in melon

SSRs and SNPs are two types of molecular markers that are widely used for gene identification, germplasm characterization, and variety fingerprinting [3, 5, 29, 30]. However, it was reported that only a small proportion of SSR/SNP loci can be selected and genotyped successfully. One study revealed that only 470 polymorphic SSRs (38.6%) were screened out from a total set of 1219 SSRs in melon [3], and another reported that only 6,406 SNPs (19.6%) had a missing rate less than 5% among the total 32,628 high-quality SNPs [8].

Furthermore, high-throughput sequencing of melon accessions identified millions of SSRs and SNPs, with an average of 60 bp in one SNP or indel in the melon genome [12]. The reason for the low success rate in PCR is that the primer design of these loci did not consider a genomic variation on the Target region and the flanking region. The main challenge is how to select representative and stable genetic loci from genome-wide variations. In this study, genome-wide perfect SSRs and perfect SNPs were selected for genetic analysis in melon. These have been successfully applied in other vegetables, such as cucumber [10, 14], eggplant [15], pepper [16], and watermelon [17].

### Development of SSR/SNP genotyping methods for melon variety identification

Variety identification is very important for the supervision of seed quality and the protection of intellectual property[26]. In the past 4 years, more than 2000 commercial melon varieties have been submitted for registration in the Chinese Ministry of Agriculture Department. Testing of these varieties poses a major challenge for the government due to the large numbers of varieties and the close similarities among them[31]. As field inspection based on morphological characters is laborious and easily influenced by the environment conditions [32], a molecular marker-based method derived from perfect SSRs/SNPs for melon variety identification was developed in this study. We also analyzed the genetic diversity and established DNA fingerprints of 259 widely cultivated varieties in China, which differs from previous studies that focused on the classification of melon germplasms [5, 6]. A significant linear correlation was found between the SSR genotyping and SNP genotyping in the discrimination of the 259 melon varieties (Fig. 3a), indicating that the molecular marker method is reliable and stable. For the first time, we constructed the pedigree of ‘Jingyu’ and ‘Jingmi’ series varieties using their DNA fingerprint, which was in accordance with their breeding history (Fig. 4). Moreover, 23 core SSRs and 40 core SNPs markers were selected, which could efficiently differentiate 99% of 259 melon commercial varieties with at least one genotypic difference. The perfect SSR and SNP loci and primers developed in this research have a broad application prospect in variety identification, variety supervision, and the protection of intellectual property rights in melon.

Population specific markers were important in variety identification which could determine the specific population by a couple of markers in an efficient way, as well as in pedigree analysis between varieties. Due to varieties were cultivated in a certain ecogeographical area adapting to the specific climatic conditions, they preserved the characteristics of landraces and formed a certain group [33]. The genomic profiling of these varieties could be used to differentiate them into different specific populations[34]. In this study, we calculated the SNP-index between the group of 58 ssp. melo and the group of 86 ssp. *agrestis* accessions with the total of 293,320 perfect SNP loci were obtained. 718 SNP of them with △SNP index values equal to 1 were designated as the population specific SNP markers, in order to distinguish the ssp. *melo* and ssp. *agrestis* varieties. Intriguingly, two SNP loci (MeSNP011and MeSNP047) were validated to identify 99% of ssp. *agrestis* varieties in the total of 259 melon varieties. This result also indicated that the few gene exchanges existing between ssp. *melo* and ssp. *agrestis* in the human breeding activities.

### Risk of genetic erosion in the ssp. agrestis varieties

It is well known that eastern China is the origin center of ssp. *agrestis,* while northwestern China is the domestication center of ssp. *melo* [13]. The history of melon cultivation in China is over 3000 years old. However, ssp. *melo* is mainly cultivated in the Xinjiang and Gansu provinces of China due to their specific environmental conditions, including a high temperature, abundant sunlight, and a large temperature difference between day and night. By contrast, the cultivation and breeding of ssp. *agrestis* are concentrated in eastern and southern China. The ssp. *melo* was initially introduced to eastern and southern China in the 1990s, and some hybrid varieties were bred from the cross of ssp. *melo* and ssp. *agrestis* melon. A far genetic distance and low level of gene flow in these two melon populations is the result of long-term geographic separation [3]. In the present study, the ssp. *agrestis* and ssp. *melo* varieties had an average *F*_*ST*_ value of 0.58, which was similar to that of the *melo* and *agrestis* groups (0.46) reported in melon germplasms [12]. Compared with ssp. *melo*, the ssp. *agrestis* varieties are subject to the risk of genetic erosion due to their narrow genetic background (Fig. 2b). We also identified an intermediate subgroup pop1C in this study which maybe the hybrid of ssp. *melo* and the ssp. *agrestis*, and some excellent melon varieties belonged to this subgroup, including ‘Xiboluotuo’, ‘Yong tian’ and ‘Yulu’. In addition, two population specific SNP loci (MeSNP011 and MeSNP047) of the ssp. *agrestis* varieties can be used as important markers in increasing the screening efficiency of hybrid melon. The comprehensive traits of the two parents used for hybridization should be complementary and distantly genetically related [35]. Therefore, increasing the gene exchange between ssp. *melo* and ssp. *agrestis* varieties is necessary and could improve the genetic diversity of the ssp. *agrestis* varieties as well as create new varieties in the future.

## Supplementary Information

**Additional file 1.**

**Additional file 2.**

**Additional file 3.**

**Additional file 4.**

## Data Availability

The datasets of genome wide perfect SNP loci are available in VegSNPDB (http://www.vegsnpdb.cn/).
