# Supplementary material for: Genetic diversity analysis and variety identification using SSR and SNP markers in melon
Source: BMC Plant Biol. 2023 Jan 18;23:39. doi: 10.1186/s12870-023-04056-7 (PMC9847184; doi:10.1186/s12870-023-04056-7)
Supplement: Supplementary file 2 — Additional file 2. [file 12870_2023_4056_MOESM2_ESM.docx]

**A perl script for selecting core set of SNP/SSR loci in variety identification**

#!/usr/bin/env perl

use strict;

use Getopt::Long ;

use File::Basename qw ( basename dirname ) ;

# 20180629 A script to calculate the saturation of accumulated marker.

sub USAGE{

print <<" Usage End.";

Description:

A script to calculate the saturation of accumulated marker.

Usage:

-i [string] input file.

-o [string] output file.

-d [int] Marker distance when selection

-nochr [Boolean] select by random,default by chromosome

-s [string] similar sample output

-h Help document

Usage End.

exit;

}

my %opts;

GetOptions( \%opts,

"i=s" , #s 字符，f浮点数，i 整数

"o=s" ,

"d=i" ,

"s=s",

"nochr!",

'h|helep+',

);

if ( ! defined $opts{i} || ! defined $opts{o} || ! defined $opts{s} || defined $opts{h} ) { ########edit

&USAGE();

}

#********************************************************************

# program start

#********************************************************************

my $Time_Start = date();

print "$0 start time : $Time_Start\n";

my $distance = defined $opts{d}? $opts{d}: 0;

open ( MARKER, $opts{i} ) || "Can not open the $opts{i}\n";

open ( OUT, ">".$opts{o} ) || "Can not open the $opts{o}\n";

my (%hash, %select, %selectChr, %saturation );

my @sample ;

my $f = 0 ; my $max = 0;

my $first_marker ;

while (<MARKER>) {

chomp;

my @a = split/\t/, $_ ;

if( $f == 0 ) {

@sample = @a; $f++;

print OUT "Saturation\t$_\n";

next ;

}

my %allele ;

for (my $i = 5; $i < @a ; $i++) {

next if($a[$i] =~/-/ );

my ($b1, $b2) = (split/\//, $a[$i])[0,1];

if($b1 eq $b2) {

$allele{$b1}++;

}

}

my $total_allele = scalar (keys %allele );

if ( $max < $total_allele ) {

$max = $total_allele; $first_marker = $_ ;

}

$hash{$a[1]}{$a[2]} = $_ ;

$selectChr{$a[1]} = 0;

}

close MARKER;

my $total_sample = scalar @sample - 5 ;

my @first = split/\t/, $first_marker ;

$select{$first[1]}{$first[2]} = $first_marker;

$selectChr{$first[1]} ++ ;

delete $hash{$first[1]}{$first[2]} ;

my %compare; my %fisrtSatu;

for (my $i = 5; $i < @first ; $i++) {

for (my $j = $i + 1 ; $j < @first ; $j++ ) {

next if ( $first[$i] ne $first[$j] && ($first[$i] ne "-" || $first[$j] ne "-") ) ;

$compare{$i}{$j} = "";

$fisrtSatu{$i}++;

$fisrtSatu{$j}++;

}

}

my $first_same =scalar( keys %fisrtSatu);

my $first_marker_saturation = 1- $first_same /$total_sample;

print "first_marker_saturation\t$first[0]\t$first_marker_saturation\n";

$saturation{$first[1]."_".$first[2]} = $first_marker_saturation;

my $f =2;

while (1) {

print $f."\n";

my $t1 = date();

Compare();

my $t2 = date();

print "$t1\t$t2\n";

$f++;

}

foreach my $id ( sort {$saturation{$a}<=>$saturation{$b}} keys %saturation ) {

my ($chr, $pos) =( split/\_/, $id )[0,1] ;

print OUT "$saturation{$id}\t$select{$chr}{$pos}\n";

#print "$saturation{$id}\t$select{$chr}{$pos}\n";

}

close OUT ;

open (SAME,">".$opts{s} ) || die $!;

if (%compare) {

my %same;

foreach my $k1 (sort keys %compare) {

foreach my $k2 (sort keys %{$compare{$k1}} ) {

if(! %same) { $same{$sample[$k1]} = "$sample[$k1]/$sample[$k2]" ; next; }

foreach my $v (sort keys %same) {

if($same{$v}=~/\Q$sample[$k1]\E/ && $same{$v}!~/\Q$sample[$k2]\E/) {

$same{$v} .= "/$sample[$k2]";

}

elsif($same{$v}!~/\Q$sample[$k1]\E/ && $same{$v}=~/\Q$sample[$k2]\E/) {

$same{$v} .= "/$sample[$k1]";

}

elsif($same{$v}!~/\Q$sample[$k1]\E/ && $same{$v}!~/\Q$sample[$k2]\E/) {

$same{$sample[$k1]} .= "$sample[$k1]/$sample[$k2]";

}

}

}

}

foreach my $s (sort keys %same) {

my @a = split/\//, $same{$s};

my $value = join "\t", @a;

print SAME "$value\n";

}

}

else {

print SAME "Those marker can differentiate all sample\n";

}

close SAME;

my $Time_End = date();

print "$0 start time : $Time_End\n";

sub date {#Time calculation subroutine

my($sec, $min, $hour, $day, $mon, $year, $wday, $yday, $isdst) = localtime(time());

$wday = $yday = $isdst = 0;

sprintf("%4d-%02d-%02d %02d:%02d:%02d", $year+1900, $mon+1, $day, $hour, $min, $sec);

}

sub Length {

my ($str) = @_ ;

while ( $str =~ /\((\w+)\)(\d+)/) {

my $string = $1 x $2;

$str =~ s/\(\w+\)\d+/$string/ ;

}

my $len = length $str;

return $len ;

}

sub Compare {

my (%addDiff, %diffSum );

foreach my $k1 ( sort {$a<=> $b} keys %compare) {

foreach my $k2 ( sort {$a<=> $b} keys %{$compare{$k1}} ) {

foreach my $chr (sort keys %hash) {

foreach my $pos ( sort {$a<=>$b} keys %{$hash{$chr}} ) {

my @str = split /\t/,$hash{$chr}{$pos} ;

my @fisrt = split/\//,$str[$k1] ;

my @second = split/\//,$str[$k2] ;

if ( $str[$k1] ne $str[$k2] && $fisrt[0] eq $fisrt[1] && $second[0] eq $second[1] && $str[$k1] ne "-" && $str[$k2] ne "-" ) {

$diffSum{$chr."_".$pos} ++;

push @{$addDiff{$chr}{$pos}}, "$k1/$k2";

}

}

}

}

}

if (! %diffSum) {

foreach my $k1 ( sort {$a<=> $b} keys %compare) {

foreach my $k2 ( sort {$a<=> $b} keys %{$compare{$k1}} ) {

foreach my $chr (sort keys %hash) {

foreach my $pos ( sort {$a<=>$b} keys %{$hash{$chr}} ) {

my @str = split /\t/,$hash{$chr}{$pos} ;

if ( $str[$k1] ne $str[$k2] && $str[$k1] ne "-" && $str[$k2] ne "-" ) {

$diffSum{$chr."_".$pos} ++;

push @{$addDiff{$chr}{$pos}}, "$k1/$k2";

}

}

}

}

}

}

last if (! %diffSum) ;

my %allowChr ;

my $min = ( sort {$a<=>$b} values %selectChr)[0] ;

foreach my $chr (keys %selectChr ) {

if(exists $opts{'nochr'}) {

$allowChr{$chr} ="";

}

else {

if ( $selectChr{$chr} == $min) {

$allowChr{$chr} ="";

}

}

}

foreach my $id (sort {$diffSum{$b}<=>$diffSum{$a}} keys %diffSum ) {

my ($chr, $pos) =( split/\_/, $id )[0,1] ;

if ( exists $allowChr{$chr} ) {

$select{$chr}{$pos} = $hash{$chr}{$pos};

$selectChr{$chr} ++ ;

delete $hash{$chr}{$pos} ;

for (my $i = 0; $i < @{$addDiff{$chr}{$pos}} ; $i++) {

my ($s1,$s2) = ( split/\//, ${$addDiff{$chr}{$pos}}[$i] )[0,1] ;

delete $compare{$s1}{$s2} ;

}

my $s = Saturation(%compare);

$saturation{$chr."_".$pos} = $s;

last ;

}

else {

$select{$chr}{$pos} = $hash{$chr}{$pos};

$selectChr{$chr} ++ ;

delete $hash{$chr}{$pos} ;

for (my $i = 0; $i < @{$addDiff{$chr}{$pos}} ; $i++) {

my ($s1,$s2) = ( split/\//, ${$addDiff{$chr}{$pos}}[$i] )[0,1] ;

delete $compare{$s1}{$s2} ;

}

my $s = Saturation(%compare);

$saturation{$chr."_".$pos} = $s;

last ;

}

}

}

sub Saturation {

my (%cal) = @_;

my %tmp;

foreach my $k1 (sort keys %cal) {

foreach my $k2 (sort keys %{$cal{$k1}} ) {

$tmp{$k1} ++;

$tmp{$k2} ++;

}

}

my $same = scalar keys %tmp;

my $saturation = 1 - $same / $total_sample ;

return $saturation;

}
