# Supplementary material for: Genetic diversity analysis and variety identification using SSR and SNP markers in melon
Source: BMC Plant Biol. 2023 Jan 18;23:39. doi: 10.1186/s12870-023-04056-7 (PMC9847184; doi:10.1186/s12870-023-04056-7)
Supplement: Supplementary file 4 — Additional file 4. [file 12870_2023_4056_MOESM4_ESM.docx]

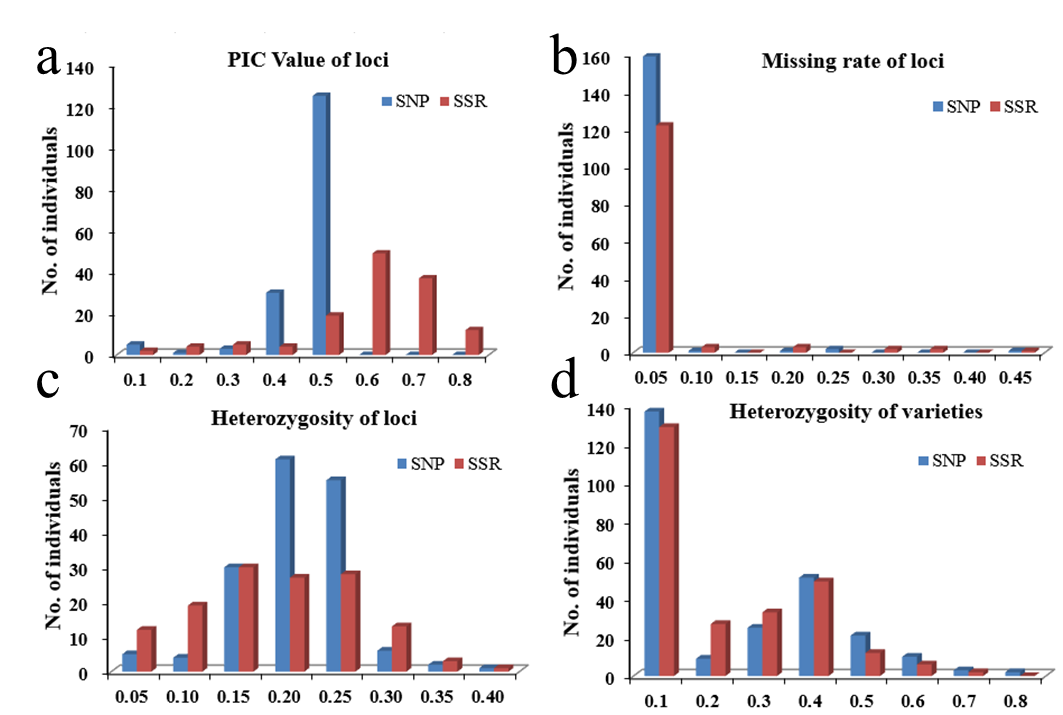


**Fig. S1** Statistics of SSR and SNP genetic variation in 259 melon varieties. (a) Polymorphism information content (PIC) value. (b) Missing rate of genotypes. (c) Heterozygosity of loci. (d) Heterozygosity of varieties.


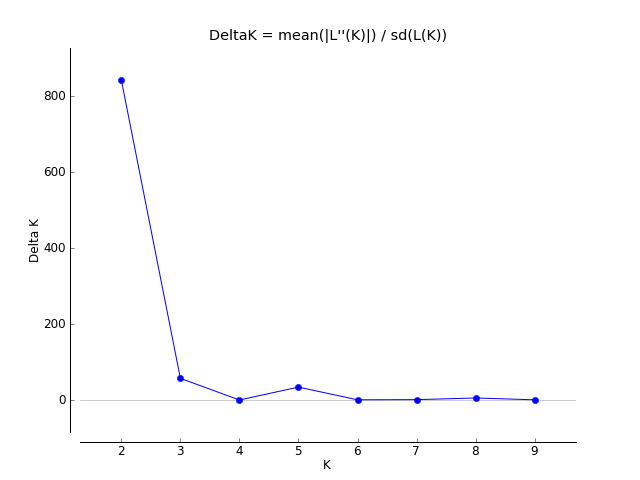


**Fig. S2** The plot of ΔK parameter from K = 2 to K =10.


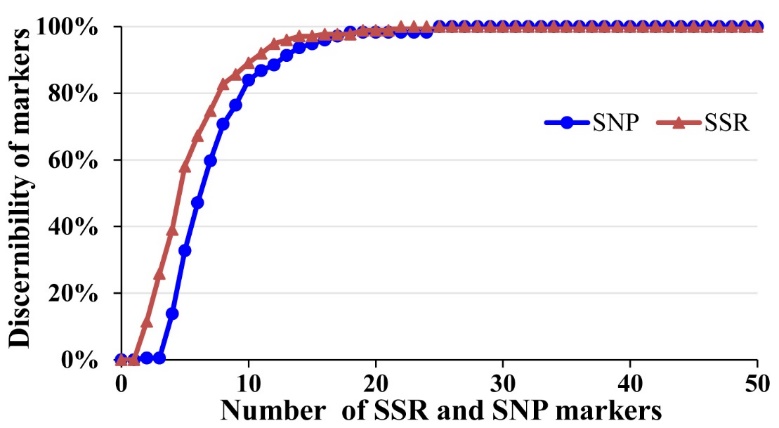


**Fig. S3** The saturation curve of 129 SSRs and 159 SNPs in 259 melon varieties distinguishment.

**
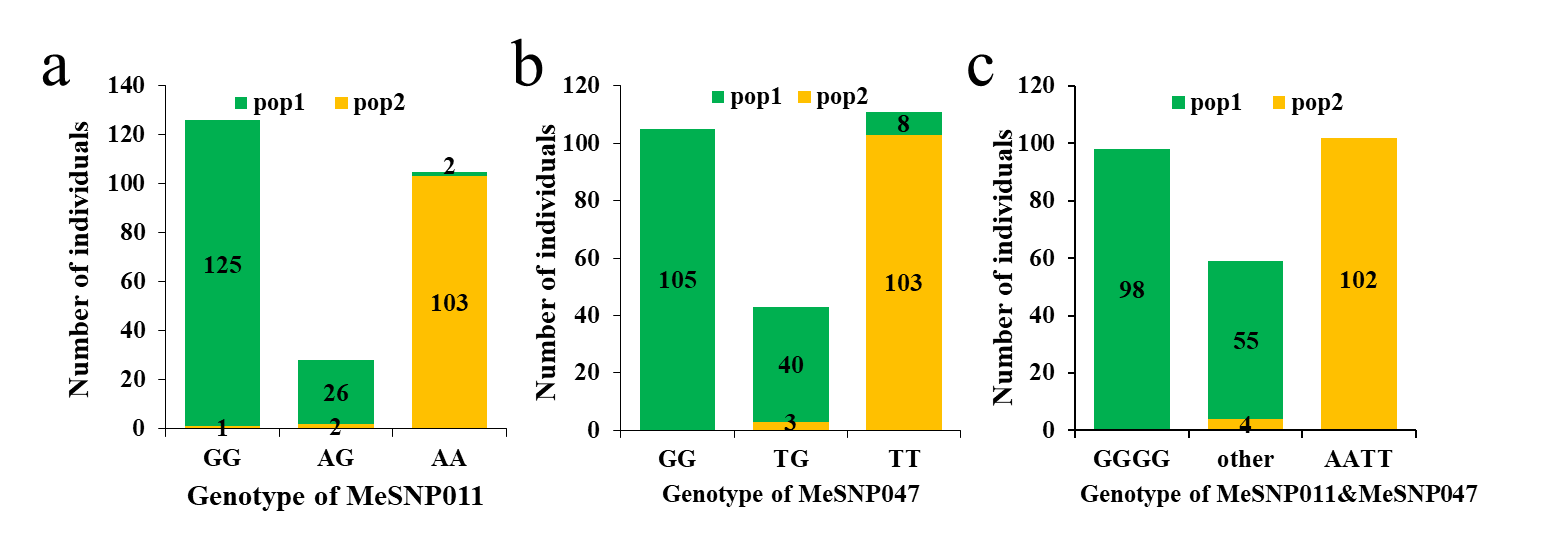
**

**Fig. S4** Number of individuals belonged to pop1 and pop2 in different genotype by MeSNP011(a), MeSNP047(b) and combination of these two markers (c).
